# The Use of Invasive Algae Species as a Source of Secondary Metabolites and Biological Activities: Spain as Case-Study

**DOI:** 10.3390/md19040178

**Published:** 2021-03-24

**Authors:** Antia G. Pereira, Maria Fraga-Corral, Paula Garcia-Oliveira, Catarina Lourenço-Lopes, Maria Carpena, Miguel A. Prieto, Jesus Simal-Gandara

**Affiliations:** 1 Nutrition and Bromatology Group, Department of Analytical and Food Chemistry, Faculty of Food Science and Technology, University of Vigo, Ourense Campus, E32004 Ourense, Spain; antia.gonzalez.pereira@uvigo.es (A.G.P.); maria.fraga.corral@hotmail.es (M.F.-C.); paula.garcia.oliveira@uvigo.es (P.G.-O.); c.lopes@uvigo.es (C.L.-L.); maria.carpena.rodriguez@uvigo.es (M.C.); 2 Centro de Investigação de Montanha (CIMO), Instituto Politécnico de Bragança, Campus de Santa Apolonia, 5300-253 Bragança, Portugal

**Keywords:** seaweeds, aquaculture feed, invasive macroalgae species, biological activity, metabolites

## Abstract

In the recent decades, algae have proven to be a source of different bioactive compounds with biological activities, which has increased the potential application of these organisms in food, cosmetic, pharmaceutical, animal feed, and other industrial sectors. On the other hand, there is a growing interest in developing effective strategies for control and/or eradication of invasive algae since they have a negative impact on marine ecosystems and in the economy of the affected zones. However, the application of control measures is usually time and resource-consuming and not profitable. Considering this context, the valorization of invasive algae species as a source of bioactive compounds for industrial applications could be a suitable strategy to reduce their population, obtaining both environmental and economic benefits. To carry out this practice, it is necessary to evaluate the chemical and the nutritional composition of the algae as well as the most efficient methods of extracting the compounds of interest. In the case of northwest Spain, five algae species are considered invasive: *Asparagopsis armata*, *Codium fragile*, *Gracilaria vermiculophylla*, *Sargassum muticum*, and *Grateulopia turuturu*. This review presents a brief description of their main bioactive compounds, biological activities, and extraction systems employed for their recovery. In addition, evidence of their beneficial properties and the possibility of use them as supplement in diets of aquaculture animals was collected to illustrate one of their possible applications.

## 1. Introduction

Invasive alien species (IAS), also known as exotic or non-native species, are plants or animals that have been introduced, intentionally or not, into regions where it is not usual to find them [1,2]. This situation often leads to negative consequences for the new host ecosystem, generally related to the community biodiversity reduction, changes in the abundance of the species and in the population’s configuration across the habitats, as well as trophic displacements that can trigger other cascade effects [3]. Spanish law 42/2007, of 13 December, on Natural Heritage and Biodiversity, defines IAS as “species that are introduced and established in an ecosystem or natural habitat, which are an agent of change and a threat to native biological diversity, either by their invasive behavior, or by the risk of genetic contamination”. IAS usually present high growth and reproduction rates, the ability to prosper in different environments, the capacity to use several food sources, and the ability to tolerate a wide range of environmental conditions. All these factors, along with the lack of natural predators, make these organisms more difficult to control and allow them to succeed in colonizing new ecosystems [3,4]. In addition, these species may feed on natural species or may carry pathogens for native organisms and even humans [5]. The invasion of non-native species also entails economic cost, which have been estimated at $1.4 trillion in the last decade [6].

Among marine IAS declared in Europe, around 20–40% are macroalgae (seaweeds) [7], a term that refers to several species of multicellular and macroscopic marine algae, including different types of Chlorophyta (green), Phaeophyta (brown), and Rhodophyta (red) macroalgae. Non-native seaweeds are particularly prone to become invasive due to their high reproductive rates, the production of toxic metabolites, and their perennial status that makes them more competitive than native species [1]. Several species periodically become a major problem, causing red tides, fouling nets, clogging waterways, and changing nutrient regimes in areas near to fisheries, aquaculture systems, and desalination facilities [1,4]. In the last years, the presence of invasive macroalgae in the northwestern marine areas of Spain has become a common problem due to growing globalization, climate change, aquaculture, fisheries, and marine tourism [8]. However, their proliferation could also offer new opportunities since the recovery of the algal biomass and their novel applications in different economic sectors could increase their added value. Obtaining natural compounds with biological properties of interest for both the food and the pharmaceutical industries is one of these possible applications. The aim of the present work is to summarize the existing knowledge about the bioactive compounds of the principal invasive species affecting the Galician coasts (northwest Spain).

## 2. Possible Exploitation of the Invasive Species

The exploitation of macroalgae is a growing industry with several applications, including human food and animal feed, biorefinery, fertilizers, production of phycocolloids, and obtaining compounds with biological properties [6,9]. Several applications are briefly discussed below.

### 2.1. Food Industry

Macroalgae have been consumed since ancient times in many countries around the world, mainly in the Asian regions. Nevertheless, their consumption has increased in the last decades in western countries, which has been attributed to the high nutritional values of macroalgae and their health benefits [10,11]. Some of the most consumed macroalgae are nori or purple laver (*Porphyra* spp.), kombu (*Laminaria japonica*), wakame (*Undaria pinnatifida*), Hiziki (*Hizikia fusiforme*), or Irish moss (*Chondrus crispus*), which can be consumed in different food formats (salads, soups, snacks, pasta, etc.) [11,12]. Still, most of them are considered an innovative niche product. Macroalgae are also widely used in the food industry to produce phycocolloids (polysaccharides of high molecular weight composed mostly of simple sugars), mainly alginates, agars, and carrageenans, which are frequently used as thickeners, stabilizers, as well as for probiotics encapsulation, gels, and water-soluble films formation [6,13]. Furthermore, diverse molecules present in algae have been shown to exert several bioactivities, such as antioxidant, anti-inflammatory, antimicrobial, and antiviral effects. These bioactive compounds (mainly proteins, polyunsaturated fatty acids, carotenoids, vitamins, and minerals) may play important roles in functional foods (e.g., dairy products, desserts, pastas, oil derivatives, or supplements) with favorable outcomes on human health [14]. Other applications of algae in the food industry include their use as colorant agents and the extraction of valuable oils (such as eicosapentaenoic acid, docosahexaenoic acid, and arachidonic acid) [15].

### 2.2. Biofuel

The development of algal biofuels (“third-generation biofuels”) has been considered an option to reduce the use of petroleum-based fuels and avoid competition between food and energy production for arable soil, since macroalgae grow in water. These organisms do not contain lignin, thus they are good substrates for biogas production in anaerobic digesters, while fermentable carbohydrates are fit for bioethanol production. Although the production of bioenergy from macroalgae is not economically feasible nowadays, several measures have been proposed to achieve a rational production cost in the future [16]. On the other hand, microalgae are considered a more suitable source to produce biodiesel due to the greater ease of controlling the life cycle and increasing the reproduction rate [17]. Microalgae biomass can be used for electricity generation or biofuel production after the lipid extraction. It has shown 80% of the average energy content of petroleum. The lipid content is highly dependent on the microalgae species and the cultivation conditions, thus not all species will be profitable, and choosing appropriate microalga strain is crucial [18]. Some microalgae used to produce biofuel are *Chlorella* spp., *Dunaliella salina, Haematococcus pluvialis*, *Spirulina platensis*, *Porphyridium cruentum*, *Microcystis aeruginosa*, and *Scenedesmus obliquus* [19].

### 2.3. Therapeutic and Cosmetic Products

The use of macroalgae for therapeutic purposes has a long history, but the search for biologically active substances from these organisms is quite recent. Numerous studies have demonstrated the biological properties of macroalgae extracts and compounds, including antioxidant, anti-inflammatory [20], antithrombotic, anticoagulant and coagulant [21], antimicrobial [22], and anticancer [23]. In addition, macroalgae have been demonstrated to exert biological properties applicable to cosmetic products, such as photo-protection, anti-aging, or anti-cellulite (Table 1). Considering this range of activities, macroalgae extracts and compounds have been considered for different pharmacologic and cosmetic products [24]. Regarding cosmetics, brown and red seaweeds are usually employed. The interest of these species lies in their content in cosmeceuticals ingredients, such as phlorotannins, polysaccharides, and carotenoid pigments [25]. These compounds are incorporated into cosmetics due to their bioactivities, their capacity to improve organoleptic properties, and their capacity to stabilize and preserve the products [26].

### 2.4. Fertilizer and Animal Feed

Currently, the negative environmental impacts of synthetic fertilizers have been identified. Thus, the use of organic fertilizers, including macroalgae, has been proposed as a suitable alternative to reduce the impact on the environment [45,46]. In fact, macroalgae have been used since ancient times as fertilizers, and several beneficial effects have been described, such as enhancement of crops growth and yield, increased resistance against abiotic and biotic stresses, or nutrient intake [46,47,48]. The biostimulant effects of macroalgae have been attributed to diverse biological compounds such as plant hormones, phlorotannins, and oligosaccharides [48].

Regarding animal feed, macroalgae have been employed for this purpose since ancient times as feed but also as nutritious supplements [49]. Several studies have evaluated the positive effects of macroalgae-enriched food, both for terrestrial animals [50] and specially in aquaculture animals [51,52,53,54].

## 3. Main Invasive Species of Northwest Spain and Their Bioactive Compounds

According to the Spanish Catalogue of IAS of Algae [55], there are 14 species of invasive seaweeds in Spain which can be divided into: (i) red species: *Acrothamnion preissii*, *Asparagopsis armata*, *Asparagopsis taxiformis*, *Grateloupia turuturu*, *Lophocladia lallemandii*, and *Womersleyella setacea*; (ii) brown species: *Gracilaria vermiculophylla*, *Sargassum muticum*, *Stypopodium schimper*, and *Undaria pinnatifida;* and (iii) green species: *Caulerpa taxifolia*, *Codium fragile*, and *Caulerpa racemosa*. In addition, there are also invasive diatoms, such as the *Didymosphenia geminata*, also known as rock snot or didymo (Table 2). However, it should be noted that this catalogue is a dynamic instrument subjected to continuous changes and updating. Most of these invasive species are originally from the Indo-Pacific Ocean (Western Australia, New Zealand, and Japan), and it is thought that they have been introduced into the Spanish coasts through the Suez Canal. Maritime traffic, ballast water, fishing nets, trade of oysters, aquaculture, and fouling are considered the main routes of dispersion [8,56,57,58].

The use of some algae (e.g., *Caulerpa racemosa*) as ornamental species in aquariums has also contributed to their proliferation [59,60]. Among these species, only five are considered invasive (*) or potentially invasive (**) in Galicia (northwest Spain): Asparagopsis armata**, *Codium fragile* subs. tomentosoides*, Grateloupia turuturu**, Sargassum muticum*, and Gracilaria vermiculophylla*. Galician waters also feature the presence of two other exotic invasive species, though they do not appear in the regulation of Real Decreto (RD) 1628/201; these are *Gymnodinium catenatum* and *Bonamia ostreae* [61]. 

For many years, non-native species of algae have been considered threats, thus a series of methods to eradicate them from non-endemic areas have been developed and optimized. However, the marine biomass, including invasive macroalgae, is currently the focus of several industries, such as pharmaceutical, food, cosmetic, and biotechnological industries, due their biological activities, e.g., antioxidant, antimicrobial, anti-inflammatory, anticancer. The aim of these industries is to revalorize invasive macroalgae as a source of extracts and compounds with industrial interest [8]. Although many studies have evaluated the biological properties of various extracts of *A. armata*, *C. fragile*, *G. turuturu*, *S. muticum*, and *G. verniculophylla*, in some cases, the bioactive compounds responsible for this activity have not yet been identified. In the following paragraphs, the current knowledge about target compounds for industrial applications and the bioactive compounds identified in the macroalgae species considered invasive in Galicia are compiled. They are also summarized in Table 3.

### 3.1. Polysaccharides

In the case of *A. armata*, the polysaccharides derived from sulfated galactans have shown strong antiviral effects against human immunodeficiency virus (HIV), inhibiting its reproduction [62]. A study confirmed the inhibition of herpes simplex virus type 1 by different extracts of numerous red algae, including *A. armata*. Although the authors did not identify the compounds involved in the activity, the good results of the water extract were attributed to water-soluble polysaccharides [63]. Mannitol has been also identified in the ethanolic extract of *A. armata*, in a concentration of 34.70 mg/100 g of dry macroalgae [64].

In the case of *C. fragile*, several bioactivities have been attributed to its sulfated polysaccharides (SPs). The administration of this type of compounds reduced the oxidative damage associated with diabetes mellitus and obesity in several animal models without any cytotoxic effect [65,66]. Recently, a study stated that SPs from *C. fragile* scavenge effectively freed radicals in vitro and suppressed the oxidative damage caused by H_2_O_2_ in Vero cell cultures and in zebrafish [67]. It has also been reported that SPs from *C. fragile* increased the coagulation time of human blood in a dose-dependent manner according to the methods activated partial thromboplastin time (APTT) [68,69], thrombin time (TT), and prothrombin time (PT) [69]. SPs from *C. fragile* inhibited HeLa cells proliferation [70] by stimulating tumor necrosis factor (TNF)-related apoptosis-inducing ligand, a promising anticancer target [71]. 

Finally, these compounds also show immune-stimulating properties in both in vitro and in vivo models. Sulfated galactan obtained from *C. fragile* stimulated murine macrophages RAW264.7 cell line, increasing the levels of nitric oxide and both pro-inflammatory and anti-inflammatory cytokines, which are fundamental for the host immune response [81,82,83]. In head kidney cells, SPs had a stimulatory effect on immune genes, including interleukin (IL)-1β, IL-8, TNF-α, interferon (IFN)-γ, and lysozyme [84]. Immuno-stimulant properties have been also observed in human peripheral blood dendritic cells and T cells, which were activated by SPs. This suggests that these compounds could be candidates for products aimed to enhance human immune system [85]. 

*S. muticum* is a source of several valuable polysaccharides, such as fucoidans, alginate, guluronic and mannuronic acids, laminarin, and their derivatives [86]. Alginate obtained from *S. muticum* has been demonstrated to possess anticancer properties, stimulating cell death in A549 cells (epithelial lung adenocarcinoma), PSN1 cells (pancreatic adenocarcinoma), HCT- 116 cells (colon carcinoma), and T98G cells (glioblastoma) [87].

Finally, G. *vermiculophylla* and *G. turuturu* are being used in the phycocolloid industry for obtaining agar and carrageenan, respectively, turning them into valuable matrixes [88,89]. Recently, polysaccharide extracts from *G. turuturu* have shown antimicrobial properties against *Escherichia coli* and *Staphylococcus aureus* [90].

### 3.2. Lipids

Starting with *A. armata*, it has been reported that these macroalgae contain some sterols such as cholesta-5,25-diene-3,24-diol, (3β,24*S*)-form [91], palmitic and stearic fatty acids, and cholestanol [64]. Recently, different crude extracts and fractions of this species were demonstrated to present antibacterial and antifouling properties. In the crude extract and most active fractions, several compounds were identified, including hexadecanoic, dodecanoic, octadecanoic, and tetradecanoic acids, which may be involved in this activity [92,93].

Regarding *C. fragile*, clerosterol (a derivative of cholesterol) was found in several extracts. This compound shows antioxidant properties, since it attenuated UVB-induced oxidative damage in human immortalized keratinocyte HaCaT cells and BALB/c mice models, reducing lipid and protein oxidation [94]. In addition, clerosterol stimulated apoptosis in A2058 human melanoma cells [95] and modulated several apoptotic factors in human leukemia cells [96]. Recently, a study observed that *C. fragile* displayed neuroprotective effects on neuroblastoma cell line SH-SY5Y. In the most bioactive fractions, several lipid compounds, among others, were identified. Although more research is needed, the authors considered that lipids are involved in the neuroprotective effect [97].

*G. vermiculophylla* contains high quantity of cholesterol (473.2 mg/kg dry weight), cholesterol derivatives, long-chain aliphatic alcohols, and monoglycerides, including 1-tetradecanol, 1-hexadecanol, 1-octadecanol, 1-eicosanol, and 1-docosanol [77]. Other lipids of great interest for nutraceutical and biotechnological industries include phospholipids, glycolipids, and eicosapentaenoic acid, present in high levels in this alga [79]. For example, three sphingolipids (gracilarioside, and gracilamides A and B) isolated from *G. vermiculophylla* (accepted name of *G. asiatica*) showed moderate cytotoxic effects against human A375-S2 melanoma cell line [98].

### 3.3. Proteins

To our knowledge, only *G. vermiculophylla* presents bioactive compounds of protein nature. This alga can absorb UV-A and UV-B radiations and decrease free radicals-induced effects, resulting from its high content in mycosporine-like amino acids [99].

### 3.4. Pigments

Siphonaxanthin from *C. fragile* has shown anticancer properties, stimulating the apoptosis of A549 lung cancer cells and modulating apoptotic factors in human leukemia cells [95,96]. Moreover, the anti-angiogenic effect of siphonaxanthin has been described in human umbilical vein endothelial cells as well as in a rat aortic ring angiogenic model [100], which suggests that this biomolecule could be an alternative to prevent pro-angiogenic diseases such as cancer. In addition, this alga also contains β-carotene [76]. 

In recent years, fucoxanthin has received a great deal of interest from the scientific community and industry due to the many beneficial health properties attributed to it, including anti-inflammatory [101]. Fucoxanthin extracted from *S. muticum* inhibited the lipopolysaccharide-induced nitric oxide production in RAW 264.7 macrophages and inhibited the expression of pro-inflammatory cytokines [102,103]. 

At industrial scale, *G. turuturu* is also used to produce R-phycoerythrin, a pink-purple pigment soluble in water present in large quantities, which presents diverse biological properties and potential industrial applications [89,104]. 

### 3.5. Vitamins

Different vitamins have been identified in the selected macroalgae, except in *A. armata*. In *C. fragile*, high levels of tocopherols have been reported (1617.6 µg/g lipid), including α, β, γ, and δ tocopherol and γ-tocotrienol [76]. *G. vermiculophylla* showed a considerable α-tocopherol content (28.4 μg/g of extract) [105]. Regarding *G. turuturu*, a chemical analysis revealed the presence of α-tocopherol and phytonadione (vitamin K1) [80]. Finally, *S. muticum* contains high amounts of α- and γ- tocopherol, 218 and 20.8 μg/g of extract, respectively [105]. 

### 3.6. Phenolic Compounds

Phenolic content has been evaluated in several species, although not all the studies have identified the target compounds. In the case of *A. armata*, phenolic content was determined by the Folin–Ciocalteu spectrophotometry method, which showed that it represented 1.13 ± 0.05% of dry weight [106]. Different extracts of *C. fragile* also contain phenolic compounds, mainly flavonoids and, to a lesser extent, tannins. These compounds showed a correlation with the antioxidant activity of the macroalgae [75]. The previous study of Farvin and Jacobsen (2013) identified several phenolic acids in both *G. vermiculophylla* aqueous extracts (gallic, protocatechuic, hydroxybenzoic, vanillic, syringic, and salicylic acids) and ethanolic extracts (gallic, protocatechuic, and gentisic acids). In correspondence with its content in phenolic compounds, a high antioxidant capacity has been demonstrated for these macroalgae according to the 2,2-Diphenyl-1-picrylhydrazyl (DPPH) and the ferric antioxidant power (FRAP) methods. In addition, *G. vermiculophylla* extracts inhibited lipid peroxidation [105]. Finally, some authors have reported the presence of phenolic compounds in *S. muticum*, including (ordered from highest to lowest concentration): hydroxybenzoic and gallic acids, *p*-hydroxybenzaldehyde, vanillic acid, 3,4-dihydroxybenzaldehyde and protocatechuic, ferulic, *p*-coumaric, caffeic, syringic, and chlorogenic acids [107]. Several bioactivities of *S. muticum*, such as antioxidant, antimicrobial, anticancer, or anti-inflammatory, have been attributed to the presence of phenolic compounds with high antioxidant capacity, particularly to phlorotannins (e.g., phloroglucinol, diphlorethol, bifuhalol), which are exclusively found in marine seaweed [78,108,109,110,111]. 

### 3.7. Other Minor Compounds

The invasive species *A. armata* presents high levels of halogenated secondary metabolites with recognized antibiotic activity [112]. They act as chemical defense against grazers and epibiota [113] and may be suitable for a wide range of applications [114,115]. For instance, the major metabolites bromoform and dibromoacetic acid, along with dibromochloromethane, bromochloroacetic acid, and dibromoacrylic acid, have shown high antifouling potential [72,73,74]. They can decrease the density of six bacteria strains on the algae surface: two marine (*Vibrio harveyii* and *V. alginolyticus*) and four biomedical strains (*Pseudomonas aeruginosa*, *Staphylococcus aureus*, *Staphylococcus epidermis*, and *Escherichia coli*) [116]. Recently, several brominated compounds, such as tribromomethanol, were found in the crude extract and fractions of *A. armata*, which showed antimicrobial antifouling potential [92,93].

A serine protease extracted from *C. fragile* was demonstrated to exert in vitro and in vivo anticoagulant and fibrinogenolytic activity [117]. Finally, it was found that *G. turuturu* contains squalene, which was reported to exert several beneficial activities [80].

## 4. Current Strategies to Obtain Bioactive Compounds from Algae

Algae have been considered as potential sources for the extraction of bioactive compounds with applications in food, cosmetic, pharmaceutical, or other industrial sectors. However, one of the most limiting steps when referring to obtaining bioactive compounds from natural sources is the extraction system, and, thus, upscale and downstream processes in the case of its industrial application [118]. Table 4 summarizes some examples of extraction techniques applied for the recovery of bioactive compounds from the studied invasive species.

For the final purpose of extracting bioactive compounds, several techniques of pretreatments and extraction have been thoroughly described. Traditionally, pretreatments consist of using hot air drying, chemical treatments with acids, salts, or surfactants. Nevertheless, novel extraction techniques (explained below) have also been successfully applied as pretreatments for algae [119].

### 4.1. Conventional Extraction Techniques

Conventional extraction techniques were deeply investigated during the past decades for their easiness of application and low requirements, but, also for this reason, they continue to be the most used [120]. As it can be seen in Table 4, the techniques that have been more frequently applied are maceration, Soxhlet, and heat assisted extraction (HAE). These methodologies are applied using different solvents, heat, and/or stirring in some cases. Moreover, in the case of Soxhlet extraction, the recircularization of the solvent during longer time periods is aimed at improving the extraction yield [121]. Additionally, heat favors the mass transfer of the bioactive compounds to the solvent through the disruption of cell walls [122].

### 4.2. Novel Extraction Techniques

On the other hand, emerging or novel techniques are also increasing as new methods directed towards a more sustainable process, with lower times and energy consumption or higher yields. Among them, some examples must be highlighted: microwave assisted extraction (MAE), ultrasounds assisted extraction (UAE), pressurized liquid assisted extraction (PLE), enzyme assisted extraction (EAE), high pressure assisted extraction (HPAE), pulsed electric field (PEF), supercritical fluid extraction (SFE), and hydrothermal liquefaction. At last, new options are being explored that combine approaches of different techniques [119]. Table 4 shows some of the examples when these techniques have been applied on invasive species. 

Considering the information collected, obtaining processes of bioactive compounds from these invasive species utilizes a wide range of conventional and novel sample preparation and extraction techniques. Once the extraction has been performed, it is necessary to characterize and quantify the compounds present in the extract. To carry out this process, the most used techniques are based on chromatographic methods [135]. These methods are regularly evolving and currently coupled to different detectors. Nuclear magnetic resonance, mass spectrometry, vibrational spectrometry, or a combination of several techniques are some of the approaches currently applied. All of them are focused on separating, detecting, characterizing, and quantifying those bioactive molecules as well as elucidating their structures and their function on the metabolic pathways they are involved in [136].

## 5. Algae as Supplement of Diets in Aquaculture

Aquaculture has grown very fast in the last decades, reaching expansion rates higher than other major food production sectors. By 2016, the aquaculture relevance as an animal protein source was underlined by its huge global production that reached nearly 80 million tons. Among the European countries, Spain is expected to reach more than 0.3 Mt of annual production [137]. This exponential growth has been prompted by the low feed conversion ratio that aquaculture species exhibit, 1.1–1.6 kg of feed/kg of edible fish, against livestock, which can reach maximum ratios of 9 kg of feed/kg of beef [138,139]. However, for the aquaculture sector to continue growing at a constant rate, the supply of nutrients and feed will have to grow at a similar rate [140]. Finding appropriate ingredients to substitute the limited marine resources generally used in aquaculture feeds has been challenging the sector for decades. Therefore, it is necessary to develop new and more sustainable food sources for aquaculture use. In this sector, macroalgae has been proposed as a possible protein source in the fish feed but also as a source of bioactive compounds, which may improve the nutritional values and exert beneficial effects on animal health, including antioxidant, antimicrobial, or positive effects on immune system [141]. Invasive algae may be possible candidates for these uses. This kind of exploitation will permit obtaining compounds from sustainable sources for industrial application while reducing the population of invasive species, providing double profit. However, several limitations of the use of macroalgae species in aquaculture feeds have been identified. For example, from a nutritional point of view, it would be necessary to eliminate compounds that may be anti-nutritive or to develop methods to reduce polysaccharides to increase the digestibility [142]. In addition, in some cases, knowledge gaps about the compounds involved in the observed effects and the mechanisms of action still persist. Therefore, the use of some species in aquaculture is still limited, and more research is necessary before their application. 

Regarding the selected invasive algae species, different examples along the scientific literature reported their beneficial effects in the nutrition of several aquaculture animals. The use of *A. armata*, under the commercial powder presentation named after Ysaline^®^100, was assessed for the development of *Sparus aurata* larvae. Among the experimental parameters analyzed—growth, survival, anti-bacterial activity, microbiota quantification, digestive capacity, stress level, and non-specific immune—the last three were not affected when *A. armata*-based feed was utilized. Besides, this diet significantly reduced the amount of Vibrionaceae present in water and larval gut and enhanced growth rate. It was suggested that mortality produced when high concentrations of *A. armata*-based feed were used will improve if lower amounts are used until 10 days after hatching, promoting a safer rearing environment [143]. Recently, extracts of *A. armata* were used to supplement the fed of the whiteleg shrimp (*Penaeus vannamei*). The results showed that the formulation increased the survival rate in presence of *Vibrio parahaemolyticus* (causative agent of acute hepatopancreatic necrosis disease) and reduced the food contamination caused by fungus [144]. 

As previously mentioned, a recent study stated the protective effect of SPs extracted from *C. fragile* against free radicals. These molecules were demonstrated to suppress the oxidative damage induced by oxygen peroxide in the main fish live model, zebrafish. Embryos at 7–9 h post-fertilization stage were incubated with different concentrations of SPs from *C. fragile* for 1 h and then exposed to the pro-oxidant agent for another 14 h. Obtained results indicated that the pre-treatment of zebrafish with *C. fragile* SPs can protect animals against oxidative stress by reducing reactive oxygen species, minimizing cell death and lipid peroxidation. This antioxidant capacity of *C. fragile* SPs can be relevant for the development of innovative fishmeal [67]. In another study, *C. fragile* SPs exerted immuno-stimulating effects on olive flounder (*Paralichthys olivaceus*), up-regulating the expression of interleukins 1β and 8, TNF-α, interferon-γ, and lysozyme genes, all of them involved in the immune response. Thus, this species could be used as feed additive to improve the immune system of the fish [84]. 

*G. vermiculophylla* has been repeatedly tested in experimental diets, especially aimed at freshwater fish species such as rainbow trout (*Oncorhynchus mykiss*). The apparent digestibility coefficient for trout of proteins and lipids from a *G. vermiculophylla* based diet was like that of the reference diets [145]. Additionally, another work in which *G. vermiculophylla* was utilized for designing experimental diets for rainbow trout demonstrated some benefits for animal health that also reflect an economical benefit for improving the quality of the finally commercialized product. The inclusion of this invasive alga in 5% doubled the flesh iodine levels, which ultimately improved the fillet color intensity and juiciness since it enhanced the carotenoid deposition, which can be also associated with a better conservation of the final product for the antioxidant properties related to carotenoid pigments [146]. In another study, the inclusion of 5% of this species in the diet of *O. mykiss* was reported to enhance the immune system of the animals by increasing lysozyme, peroxidase, and complementing system activities, which play a key role in the defense against pathogens [147]. Finally, the effect of supplementation of heat-treated *G. vermiculophylla* was evaluated in gilthead sea bream (*Sparus aurata*) submitted to acute hypoxia and successive recovery. Compared to the control, the dietary inclusion of the macroalgae reduced the antioxidant stress caused by the hypoxia, and the survival rate was higher [148]. More recently, the immunomodulatory effect of *G. vermiculophylla* has been evaluated in the shrimp *Litopenaeus vannamei*. Co-culture with diverse macroalgae species (including *G. vermiculophylla)* improved the immune response of the shrimps against the pathogen *V. parahaemolyticus* and white spot virus, increasing the production of hemocytes and the activity of superoxide dismutase (SOD) and catalase (CAT) compared to control [149]. 

Very scarce information regarding the development of experimental diets formulated with *S. muticum* has been found. However, at least one study performed its inclusion and tested its effect in African catfish, *Clarias gariepinus.* As in previous works, they added 5% of alga and fed animals for 12 weeks. In the skin of fish fed with probiotics diet, an improved glutathione *S*-transferase (GST) and SOD activity and less CAT activity were recorded, whereas in the livers from fishes fed with *S. muticum*, a better oxidative status with improved GST and CAT activities were displayed. This positive effect on antioxidant enzyme activity has been suggested to ultimately improve the resistance of animals against bacterial infections [150]. Other species belonging to the *Sargassum* genus have been described as immunomodulators and growth promoters for great sturgeon (*Huso huso*) and as immunobooster for shrimp (*Fenneropenaeus chinensis*) to which they also provide specific resistance to vibriosis [151,152].

Finally, experimental diets aimed to feed cultivated hybrid abalone cross (*Haliotis rubra* and *Haliotis laevigata*) were designed using several macroalgae, i.e., *G. turuturu* together with *Ulva australis* and/or *U. laetevirens*. Treatment applied for 12 weeks period provided a significant higher growth rate of abalone in terms of length and weight. Besides, it improved abalone health and its nutritional composition, since animals showed, by the end of the assay, tissues with higher carbohydrate/protein ratio, ash content, and lower lipid amount [153]. Other studies in which *G. turuturu* mixed with *P. palmata* was used as feed for the European abalone *Haliotis tuberculate* demonstrated that the combination of algae did not produce animals’ mortality, and it improved growth rates (in length and weight) while increasing the final content of lipid in the abalone [154]. Besides, in another work, the capacity of *G. turuturu* was underlined for inhibiting, in a quantity of 16%, the growth of the main pathogen of the *H. tuberculata*, that is, *Vibrio harveyi* [130]. Therefore, the inclusion of this invasive alga in experimental diets may provide nutritional value to abalone but also antibacterial activity which ultimately reduces mortalities.

## 6. Future Perspectives and Conclusions

According to the compiled studies, *Asparagopsis armata*, *Codium fragile* subs. *tomentosoides*, *Grateloupia turuturu*, *Sargassum muticum*, and *Gracilaria vermiculophylla* can be considered as alternative sources of bioactive compounds which could be further used for industrial applications. Thus, revalorization strategies will make it possible to obtain new compounds from sustainable sources but also reduce the population of invasive species, generating a double benefit. Nevertheless, two key concerns limit their further use. From the scientific and the technological points of view, more research is still required to increase the profitability of the extraction process. Therefore, the applicability of different techniques needs to be further investigated to assess which is the most favorable process, comparing both conventional and modern extraction techniques. In addition, in some cases, it is still necessary to identify the specific compounds responsible for the observed activities and to determine their action mechanisms. Nevertheless, the development of invasive algae harvesting methods generates a series of drawbacks. The main one is that the revalorization of invasive algae could lead to an increase of their populations instead of eliminating them due to the economic benefits that could be obtained from their use. In fact, this economic revenue would not be difficult to achieve, since these invasive algae are often characterized by a high reproductive rate. Considering this drawback, the collection of invasive species should be subjected to a strict policy. A principle that should be considered is that the only legal collectors of invasive algae should be those companies whose activity is reduced by the presence of these organisms (e.g., shellfish catchers/farmers, inshore fishermen, diving companies, etc.). This would prevent the harvesters themselves from “planting” more invasive algae to further increase their profits. 

## Figures and Tables

**Table 1:** Properties and applications of extracts and compounds isolated from algae in the cosmetic field.

Treatment	Specie	Compound	Result	Ref.
Skin aging	*Alaria esculenta*	Extract	Decline the amount of progerin in aged fibroblasts at the lowest tested concentration (not for younger cells)	[27]
	*Phaeodactylum tricornutum*	Ethanol extract	Protecting the skin from the adverse effects of UV exposure; preventing and/or delaying the appearance of skin aging effects	[28]
	*Hizikia fusiformis*	Fucosterol	Inhibit metalloproteinase-1 expression	[29]
	*Ecklonia stolonifera*	Phlorotannins	Inhibit metalloproteinase-1 expression	[30]
Sunscreen	*Halidrys siliquosa*	Phlorotannins	UV-filter activity	[31]
	*Brown seaweeds*	Phlorotannins	Protective effect against photo-oxidative stress	[32]
	*Corallina pilulifera*	Phenolic compounds	Anti-photoaging activity and inhibition of matrix metalloproteinase	[33]
	*Sargassum* spp.	Fucoxanthin	Protective effect on UV-B induced cell damage	[34]
	*Sargassum confusum*	Fucoidan	Suppress photo-oxidative stress and skin barrier perturbation in UVB-induced human keratinocytes	[35]
	*Macrocystis pyrifera*, *Porphyra columbina*	Acetone extracts	In vivo UVB-photoprotective activity	[36]
Moisturizer	*Fucus vesiculosus*	Fucoidan	Inhibition of hyaluronidase enzyme	[37]
	*Laminaria japonica*	5% water:propylene glycol (50:50) extracts	Hydration with the alga extract increased by 14.44% compared with a placebo	[38]
	*Rhizoclonium hieroglyphicum*	Polysaccharides and amino acids	Similar moisturizing effects to hyaluronic acid and glycerin	[39]
Whitening	*Nannochloropsis oculata*	Zeaxanthin	Antityrosinase activity	[40]
	*Laminaria japonica*	Fucoxanthin	Antityrosinase activity	[41]
	*Arthrospira platensis*	Ethanol extract	Antityrosinase activity	[42]
Hair care	*Chlorella* spp.	Intact microalga cells	Soften and make flexible both skin and hair	[43]
	*Ecklonia cava*	Dioxinodehydroeckol	Promote hair growth	[44]

**Table 2:** Invasive algae species in Spain: taxonomy, origin, geographical distribution, and principal uses.

Specie	Taxonomy	Native Distribution	Distribution in Spain	Other Regions in Which They are Invasive	Principal Uses
Red species					
*Acrothamnion preissii*	Phylum: *Rhodophyta* Class: *Florideophyceae* Orden: *Ceramiales* Family: *Ceramiaceae*	Western Australia	All Spain	Temperate coastlines on the Pacific coast of North America and western coasts of Europe	- Unknown
*Asparagopsis armata*	Phylum: *Rhodophyta* Class: *Florideophyceae* Orden: *Bonnemaisoniales* Family: *Bonnemaisoniaceae*	Indo-Pacific Ocean	All Spain	Mediterranean, Portugal, and Ireland	- Pharmaceutical potential as antibiotic
*Asparagopsis taxiformis*	Phylum: *Rodophyta* Class: *Rhodoplayceae* Orden: *Nemaliales* Family: *Bonnemaisoniaceae*	Australia and New Zealand	Except Canarias	Portugal	- Human consumption - Antifungal
*Grateloupia turuturu*	Phylum: *Rhodophyta* Class: *Florideophyaceae* Orden: *Halymeniales* Family: *Halymeniaceae*	Pacific Ocean	All Spain	North America, Europe, and Oceania	- Human consumption - Fertilizer
*Lophocladia lallemandii*	Phylum: *Rhodophyta* Class: *Florideophyceae* Order: *Ceramiales* Family: *Rhodomelaceae*	Indo-Pacific Ocean	All Spain	Mediterranean	- Unknown
*Womersleyella setacea*	Phylum: *Rhodophyta* Class: *Rhodophyceae* Order: *Ceramiales* Family: *Rhodomelaceae*	Indo-Pacific Ocean	All Spain	Mediterranean	- Unknown
Brown species					
*Gracilaria vermiculophylla*	Phylum: *Rhodophyta* Class: *Florideophyceae* Orden: *Gracilariales* Family: *Gracilariaceae*	North-east Pacific	All Spain	Europe and North America	- Animal feed - Biofuels - Fertilizer - Human consumption
*Sargassum muticum*	Phylum: *Ochrophyta* Class: *Phaeophyceae* Order: *Fucales* Family: *Sargassaceae*	Indo-Pacific Ocean	All Spain	Pacific Coast of North America, North Sea, Portugal, and the Mediterranean	- Animal feed - Food additive - Pesticide
*Stypopodium schimperi*	Phylum: *Ochrophyta* Class: *Phaeophyceae* Order: *Dictyotales* Family: *Dictyotaceae*	Indo-Pacific Ocean and Red Sea	All Spain	Africa and Southwest Asia	- Unknown
*Undaria pinnatifida*	Phylum: *Heterokontophyta* Class: *Phaeophyceae* Order: *Laminariales* Family: *Alariaceae*	Asia	All Spain	Europe	- Human consumption - Animal feed
Green species					
*Caulerpa taxifolia*	Phylum: Chlorophyta Class: Bryopsidophyceae Orden: Bryopsidales Family: Caulerpaceae	Tropical area	All Spain	Mediterranean, California, and southern Australia	- Laboratory use
*Codium fragile*	Phylum: Chlorophyta Class: Chlorophyceae Orden: Codiales Family: Codiaceae	North of the Pacific Ocean and coast of Japan	All Spain	Widespread in the Mediterranean	- Human consumption
*Caulerpa racemosa*	Phylum: Chlorophyta Class: Bryopsidophyceae Orden: Bryopsidales Family: Caulerpaceae	Tropical areas	Except Canarias	Mediterranean: from Spain to Turkey	- Human consumption
Diatoms					
*Didymosphenia geminata*	Phylum: Ochrophyta Class: Bacillariophyceae Orden: Cymbellales Family: Gomphonemataceae	Boreal and alpine regions of North America and Northern Europe	All Spain	New Zealand and Patagonia, South America	- Ornamental

**Table 3:** Main compounds and bioactive compounds reported for the invasive macroalgae in northwest Spain.

Bioactive compounds	Invasive Macroalgae				
	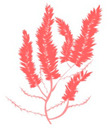	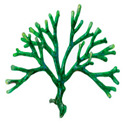	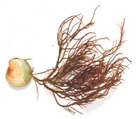	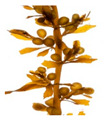	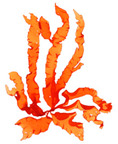
	*Asparagopsis armata*	*Codium fragile*	*Gracilaria vermiculophylla*	*Sargassum muticum*	*Grateloupia turuturu*
Polysaccharides	Sulphated galactan derivatives, Mannitol	Sulphated polysaccharides		Fucoidans, Alginate, Glucuronic acid, Mannuronic acid, Laminarin	
Lipids	Cholestanol, Cholesta-5,25-diene-3,24 -diol, Palmitic acid, Stearic acid	Clerosterol	Cholesterol, 1-tetradecanol, 1-hexadecanol, 1-octadecanol, 1-eicosanol, 1-docosanol, Sterols, Monoacylglycerol	α -Linolenic acid	Phospholipids, Glycolipids, Eicosapentaenoic acid
Proteins			Mycrosporine-like aminoacids*		
Pigments		β-carotene, Siphonaxanthin		Fucoxanthin	R-phycoerythrin
Vitamins		α, β, γ, δ-tocopherol, γ-tocotrienol	α-tocopherol	α, γ-tocopherol	α-tocopherol, Phytonadione (vitamin K1)
Phenolic compounds	Not specified	Flavonoids, tannins	Gallic acid, Protocatechuic acid, Gentisic acid, Hydroxybenzoic acid, vVnillic acid, Syringic acid	Hydroxybenzoic acid, Gallic acid, Vanillic acid, Protocatechuic acid, Caffeic acid, Syringic acid, Chlorogenic acid, Coumaric acid, Phlorotannins, Fuhalols, Phlorethols, Hydroxyfuhalols, Monofuhalol A,	
Other compounds	Halogenated compounds, *Halogenated ketones*, 1,1-dibromo-3-iodo-2- propanone, 1,3-dibromo-2- propanone, 1,3-dibromo-1-chloro-2- propanone (±) form, *Halogenated carboxylic acids*, Dibromoacetic acid, Bromochloroacetic acid, Dibromoacrylic acid, *Halogenated alkanes*, Bromoform, Dibromochloromethane	Serine protease	Long chain aliphatic alcohols	Tetrapernyltaluquinol meroterpenoid with a chrome moiety	Squalene
Reference	[72,73,74]	[75,76]	[77]	[78]	[79,80]

**Table 4:** Extraction techniques for obtaining bioactive compounds from the invasive macroalgae in northwest Spain.

Method	Conditions	Compounds	Activities	Model/Assay	Ref.
*Asparagopsis armata*					
Soxhlet	Chloroform-methanol (3:2), dichloromethane (100%), methanol (100%), and water (100%), 8 h	-	Anti-Herpes Simplex Virus and cytotoxicity	Neutral red dye method on Vero cells.	[63]
Mac	Hexane, dichloromethane, and ethanol	Halogenated compounds	Antiprotozoal	*Leishmania donovani* promastigotes cultures	[123]
Mac	0.025 g/mL; methanol, 16 h, 20 °C	Phenolic compounds	Antioxidant and neuroprotective	DPPH, CCA, ICA. AChE, BuChE, TYRO inhibition. In vivo MTT assay on SH-SY5Y cells on H_2_O_2_ induced cytotoxicity.	[124]
HAE	0.04 g/mL; distilled water, 5 h, 96 °C	Polysaccharides	Anti-HIV	Human immunodeficiency virus (HIV) induced syncytium formation on MT4 cells.	[62]
PLE	Dichloromethane methanol (1:1; v:v); 75 °C, 1500 psi, 7 min (×2)	Phenolic compounds	Antioxidant and cytotoxicity	Radical-scavenging activity (DPPH). Reducing activity. Daudi, Jurkat and K562 cell lines.	[106]
*Codium fragile*					
Mac	80% methanol (×3). Butanol and ethyl-acetate fractions.	Clerosterol	Antioxidant and anti-inflammatory	In vivo MTT assay on human keratinocyte HaCaT cells irradiated with UVB and BALB/c mice models. Expression of pro-inflammatory proteins and mediators	[94]
Mac	Hexane, ethyl, and methanol (×3)	-	Antioxidant and anti-hypertensive	DPPH and ABTS inhibition In vitro ACE inhibitory assay	[75]
Mac	80% methanol	-	Anti-inflammatory	Lipopolysaccharide-stimulated RAW 264.7	[125]
Mac	80% methanol	-	Anti-cancer	Human breast cancer cell line MDA-MB-231	[126]
HAE	0.02 g/mL; water, 12 h, 60 °C	Polysaccharides	Anticoagulant	APTT assay on human blood	[68]
HAE	10 vol, distilled water, 1 h, 95 °C	-	Anti-inflammatory and anti-edema	LPS-stimulated RAW 264.7 and carrageenan-induced paw edema in male Sprague-Dawley rats.	[127]
HAE	Ethanol 96% (*v*/*v*), 3 h, 70 °C (×3)	-	Anti-inflammatory	LPS-stimulated RAW 264.7.	[128]
HAE	Distilled water, 4 h, 90 °C.	-	Anti-inflammatory, alleviation of cartilage destruction	Primary chondrocytes cells, osteoarthritis rat model.	[129]
*Gracilaria vermiculophylla*					
Mac	0.1 g/mL; water or ethanol, 96%, 12 h, room temperature.	Phenolic compounds	Antioxidant	In vitro assays (DPPH, FRAP, ferrous ion-chelating) and liposome model system.	[105]
Soxhlet	0.3 g/mL; ethyl acetate; 72 h.	-	Antimicrobial	Strains of *S. enteritidis*, *P. Aeruginosa* and *L. innocua*	[121]
*Grateloupia turututu*					
S/L	1/20 ratio (*w*/*v*), water, 20 min, phosphate buffer (20 mM, pH 7.1)	-	Antibacterial	European abalone pathogen *Vibrio harveyi*	[130]
*Sargassum muticum*					
Mac	0.01 g/mL; 80% methanol, 24 h, RT.	Fucoxanthin	Anti-inflammatory	LPS-stimulated RAW 264.7 macrophages	[103]
Mac	0.1 g/mL; Water or ethanol, 96%, 12 h, RT.	Phenolic compounds	Antioxidant	In vitro assays (DPPH, FRAP, ferrous ion-chelating) and liposome model system	[105]
Mac	Dichloromethane or methanol, 1:4 (*w*/*v*), 12 h.	Phenolic compounds	Antioxidant and cytoprotective effect	In vitro assays (DPPH and ORAC) Protective effect on MCF-7 cells	[131]
HAE	Methanol:water (1:10), 3 h, 65 °C (×3)	Chromane meroterpenoid	Photodamage attenuation	Human dermal fibroblasts	[132]
SFE	CO_2_, 10% ethanol, 15.2 MPa, 60 °C, 90 min (static)	-	Antioxidant	Not reported	[133]
PLE	Ethanol:water (95:5); 160 °C, 10.3 MPa, 20 min (×2)	Phlorotannins	Antiproliferative	HT-29 adenocarcinoma colon cancer cells	[134]
UAE	Water at S/L ratio of 1:20; 5–30 min, RT (25 °C), 5 A, 150 W and 40 Hz.	Alginate	Cytotoxic effect	A549, HCT- 116, PSN1, and T98G cells	[87]
Autohydrolisis	96% ethanol	-	Antioxidant, anti-inflammatory and anti-irritant	In vitro assays (FRAP, DPPH and ABTS). Reconstructed human epidermis test method. Irritability assays with the Episkin test.	[108]
Autohydrolisis	RT, formaldehyde 1% (15 h), sulfuric acid 0.2 N (4 h), and sodium carbonate 1% (15 h).	Phlorotannins	Anti-tumor and anti-inflammatory	A549, HCT-116, PSN1, and T98G cells. Neutrophils’ oxidative burst oxidation of luminol.	[78]

Extraction method: PLE: pressurized liquid extraction; S/L: solid–liquid; SFE: supercritical fluid extraction; UAE: ultrasound assisted extraction; Mac: maceration; RT: room temperature. Assays: DPPH: 1,1-Diphenyl-2-picrylhydrazyl; ABTS: 2,2′-azino-bis(3-ethylbenzothiazoline-6-sulfonic acid; CCA: copper chelating activity; ICA: iron chelating activity; AChE: acetylcholinesterase; BuChE: butyrylcholinesterase; TYRO: tyrosinase; ACE: angiotensin converting enzyme; APTT: activated partial thromboplastin Time; ORAC: oxygen radical absorbent capacity; FRAP: ferric antioxidant power. Cell lines: Vero: African green monkey kidney cell line; MT4: leukemia cell line; HaCaT: aneuploid immortal keratinocyte cell line; RAW 264.7: murine macrophage cell line; MCF-7: human breast cancer cell line; A549: adenocarcinomic human alveolar basal epithelial cells; HCT-116: human colon cancer cell line; PSN1: human pancreatic cancer cell line; T98G: glioblastoma cell line.

## Data Availability

Not applicable.
